# Knowledge, Attitude, and Practices on Antimicrobial Use and Antimicrobial Resistance among Poultry Drug and Feed Sellers in Bangladesh

**DOI:** 10.3390/vetsci8060111

**Published:** 2021-06-15

**Authors:** Md. Abul Kalam, Md. Abdul Alim, Shahanaj Shano, Md. Raihan Khan Nayem, Md. Rahim Badsha, Md. Abdullah Al Mamun, Ashraful Hoque, Abu Zubayer Tanzin, Shahneaz Ali Khan, Ariful Islam, Md. Mazharul Islam, Mohammad Mahmudul Hassan

**Affiliations:** 1 Helen Keller International, Dhaka 1212, Bangladesh; a.kalam724@gmail.com; 2 Faculty of Veterinary Medicine, Chattogram Veterinary and Animal Sciences University, Chattogram 4225, Bangladesh; maalim85@gmail.com (M.A.A.); raihannayem@gmail.com (M.R.K.N.); mabdullahal8@gmail.com (M.A.A.M.); ashraful.cvasu@gmail.com (A.H.); tanjin04@gmail.com (A.Z.T.); shahneazbat@gmail.com (S.A.K.); 3 EcoHealth Alliance, New York, NY 10001-2320, USA; shahanajshano@gmail.com (S.S.); arif@ecohealthalliance.org (A.I.); 4 Institute of Epidemiology, Disease Control and Research (IEDCR), Dhaka 1212, Bangladesh; 5 Faculty of Food Science and Technology, Chattogram Veterinary and Animal Sciences University, Chattogram 4225, Bangladesh; mrbadsha1322@gmail.com; 6 Centre for Integrative Ecology, School of Life and Environmental Science, Deakin University, Geelong Campus, Geelong, VIC 3216, Australia; 7 Department of Animal Resources, Ministry of Municipality and Environment, Doha P.O. Box 35081, Qatar; mmmohammed@mme.gov.qa

**Keywords:** antimicrobial use, antimicrobial resistance, poultry feed seller, poultry drug seller, knowledge, attitude, practice

## Abstract

Poultry production has boomed in Bangladesh in recent years. The poultry sector has contributed significantly to meet the increased demand for animal source proteins in the country. However, increased use of antimicrobials appeared to be a significant threat to food safety in the poultry sector. The poultry drug and feed sellers are at the frontline position involving selecting and delivering the antimicrobials to the poultry farmers. Studies assessing the poultry drug and feed sellers’ knowledge, attitudes, and practices (KAPs) are limited. The current study aimed to assess the community poultry drug and feed sellers’ KAPs of antimicrobial use (AMU) and antimicrobial resistance (AMR) in some selected areas of Bangladesh. We determined the respondents’ (drug and the feed sellers) KAPs of AMU and AMR using a tested and paper-based questionnaire. The study demonstrated that most respondents have insufficient knowledge, less positive attitudes, and inappropriate practices regarding AMU and AMR. The factor score analysis further showed that the type of respondents and their years of experience, level of education, and training on the drug were the significant factors impacting the current knowledge, attitudes, and practices of AMU and AMR. The adjusted logistic regression analysis revealed that the drug sellers who completed their education up to 12th grade and had training on the drug had adequate knowledge of AMU and AMR. The data also showed that the drug sellers belong to the age group 31–35 and 36–40 years and who completed 12th grade had good attitudes on the same. Likewise, the analysis further determined that drug sellers belonging to the age category 18–25 and 26–30 years, and interestingly, the respondents who completed education up to 12th grade, had better practices. Spearman’s rank-order correlation revealed a positive association between each pair of the KAPs scores for the respondents. The correlation was fair between knowledge–attitudes, knowledge–practices, and attitudes–practices. Based on the current study results, we recommend educational interventions and appropriate training for the poultry drug and feed sellers to raise awareness and to upgrade their current knowledge on the appropriate use of antimicrobials. This will ultimately lead to reducing the chances of developing AMR in the poultry sectors of the country.

## 1. Introduction

Poultry meat and egg production have boomed and predominantly crossed the expected production level over the past decades in the South East Asian countries, including Bangladesh [1]. Bangladesh produced a substantial amount of protein sources through its improved poultry production channel during 2017 and 2018. About 150,000 farms across the country produced 3.38 billion poultry, 7.26 million tons of meat, and 155.2 billion eggs during this period [2], contributing to animal source protein for the people. Among poultry species, 90% of the protein derived from chickens, followed by ducks (8%) and other species, such as quail, pigeons, and geese (2%) [3]. Increased numbers of commercial farms, modern technology, and rearing high-yielding poultry variety have been implemented to increase production in recent years. The poultry sector is considered a vital part of the economy in Bangladesh in the recent period, overburdening the antimicrobial resistance (AMR) issues [4]. AMR in antibiotics is crucial in poultry production from therapeutic and preventive aspects [5]. Unfortunately, inappropriate and indiscriminate use of antimicrobials (AMU) leads to the emergence, transmission, and persistence of AMR in the agricultural production system [6]. In this process, microbes acquire the ability to tolerate one or more antimicrobials that they rely on to treat microbial infections [7]. Antimicrobial residues in food products have the consequence of developing AMR in humans and animals [8]. Antimicrobial residues or their metabolites and/or associated impurities in any edible portion of an animal product [9,10,11,12,13] might be the cause of AMR problems in humans. These residue concentrations become detrimental if they cross the standard residue limits into the human food chain [14].

As a developing country, Bangladesh is vulnerable to developing AMR. Parallel to its snowballing livestock sector, irrational use of antimicrobials is also increasing in the livestock production system. Antimicrobials such as amoxicillin, tetracycline, oxytetracycline, ciprofloxacin, enrofloxacin, sulphadiazine, and trimethoprim are frequently used on poultry farms in Bangladesh [15]. Besides infectious cases, these antimicrobials are commonly used as growth promoters in poultry feed formulations to increase production [16]. The worst thing is that, most of the time, these antimicrobials are administered without seeking prescription from a registered veterinarian [17]. There is a risk of environmental microorganisms getting exposed to those antimicrobial drugs in the human food chain. Furthermore, this could develop a depot of resistance genes for humans and other animals [18]. Therefore, animal health care providers are essential personnel to overcome this current situation [19]. Feed and drug-producing companies, their agents, drug sellers, feed sellers, veterinarians, and farmers could be vital in this battle in the production procedure through their consciousness and perceptions. The government of Bangladesh has already enforced laws and control measures for the rational use of antimicrobial drugs [20]. Unfortunately, there are breaches of these laws by different feed industries to strive with this competitive market. Feed producing companies add different antimicrobial agents in poultry feed so that their product performs the best and they can compete with other companies [21]. The drug companies are producing some antimicrobial drugs banned for poultry or other animals in the country [22]. Their agents promote their products to feed sellers, drug sellers, quacks, and even farmers to use them unnecessarily [23]. As the poultry farmers have to rely on the feed sellers for many aspects of poultry production, therefore, the drug and feed sellers often suggest antimicrobials to the farmers. Most often, they suggest using different antimicrobials with a prescribed schedule for the whole flock, although there is no infection sign yet. Farmers further consider the drug sellers directly to better produce their small-scale poultry farming [24]. Sometimes, these feed and drug sellers themselves alter the antimicrobial drugs and their doses prescribed by the registered veterinarians. However, the feed and drug sellers frequently advise antimicrobial drugs to the farmer without prescription and any definite pharmacology knowledge, such as dose, duration, interval, maintaining the withdrawal period, and residual effects [25]. In this way, they violate laws and provide inappropriate suggestions of antimicrobials to the farm level, resulting in an increased AMR burden for the country [24]. The farmers become enthusiastic as their production increases through feed and drug sellers [26]. These stakeholders (drug and feed sellers) have minimal knowledge of antimicrobial residue [27]. Human’s consumption of poultry products with low-dose antimicrobials residue may impact on commensals and other bacteria to develop AMR [28,29,30]. In such cases, the invention of advanced antimicrobials is mandatory to face the new superbugs [31]. Health issues on AMR for humans, including the high cost of treating infectious disease, transmitting AMR bacteria into other food sources via poultry products, is essential [12,18,32]. Improvement of knowledge, attitude, and practices (KAPs) of AMU and AMR of poultry production channel, especially from the feed and drug sellers’ perspective is essential to minimize AMR issues in Bangladesh. There is no similar type of study conducted so far on the same issue to the best of our knowledge. Therefore, this study was undertaken to identify the KAPs of AMU and AMR of feed and drug sellers and propose some interventions required for minimizing AMR threats in the food chain via the poultry production system of the country.

## 2. Materials and Methods

### 2.1. Study Period and Areas

**Figure 1:**
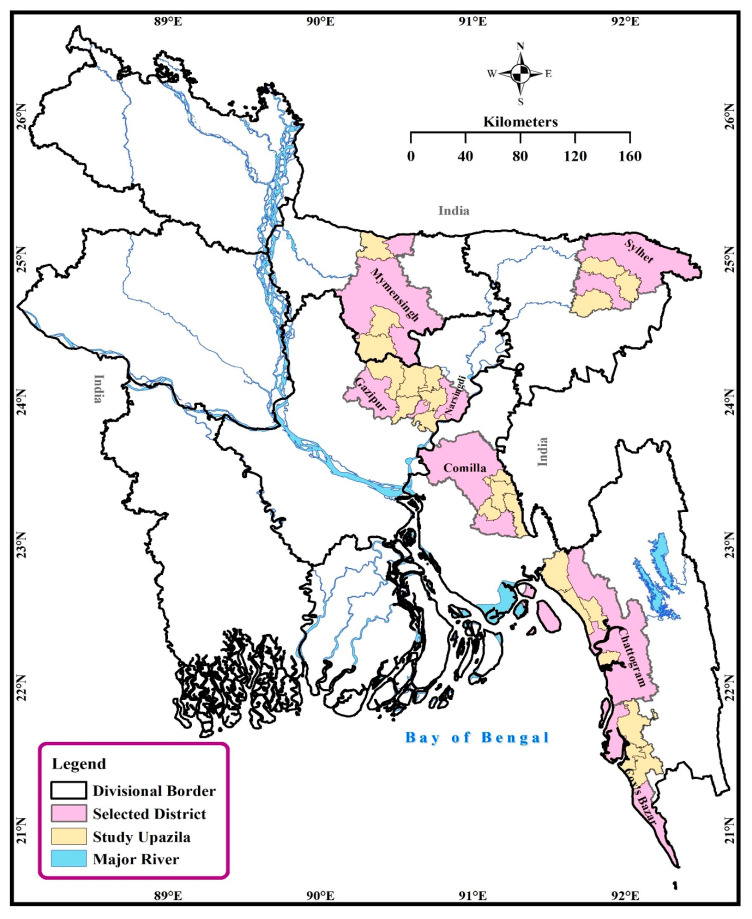
Map depicting the studied district (color: pinkish) and Upazila (smallest administrative boundaries; color: yellowish) of Bangladesh.

We conducted this survey for a period of 6 (six) months, starting from October 2019 to March 2020. We considered 22 (twenty-two) Upazilas of 7 (seven) districts of Bangladesh (Figure 1). A Upazila (a sub-district) is the lowest administrative boundary of a district in Bangladesh. The study sites were selected based on the availability of poultry drug and feed shops and the area with a higher number of poultry farms.

### 2.2. Study Area and Sampling

A cross-sectional quantitative survey on community poultry drug and feed sellers was conducted in different districts of Bangladesh. We collected data from 220 respondents (*n* = 110 for drug and feed sellers, respectively) from 7 (seven) districts (Chattogram, *n* = 40; Cumilla, *n* = 30; Cox’s Bazaar, *n* = 30; Gazipur, *n* = 30; Mymensingh, *n* = 30; Narsingdi, *n* = 30; and Sylhet *n* = 30) (Table S1, Table 1). The sample size was calculated using the Raosoft sample volume calculation method based on a 5% error rate, 85% reliability level, and 50% response distribution [33]. As there were no similar types of studies conducted as such in Bangladesh, we assumed 50% of the respondents would have proper knowledge on AMU, and AMR, added 5% to the sample to address the non-response rate. Thus, a minimum sample size of 208 is necessary for conducting the study. All the data were collected using a pre-designed paper-based questionnaire.

### 2.3. Data Instrument and Collection

**Figure 2:**
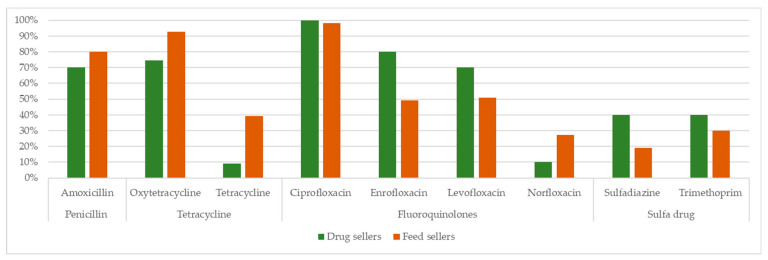
Comparative scenario of major antimicrobials practices by poultry drug and feed sellers.

**Figure 3:**
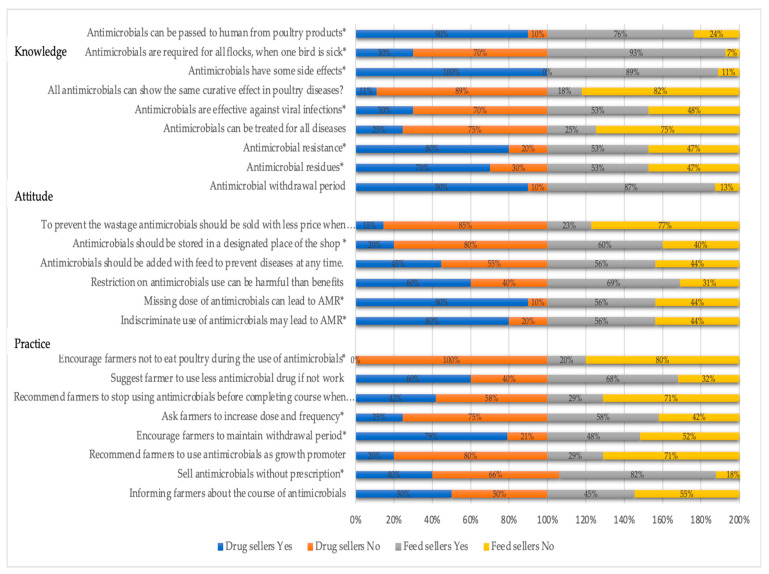
Knowledge, attitudes, and practices related to antimicrobial use (AMU) and antimicrobial resistance (AMR). * *p*-value < 0.05.

The questionnaire comprised five different sections. Demographic information such as age (in years), type of respondent (drug or feed seller), level of education, years of experience in selling drugs and feeds, and any training received on drug-related information was obtained in the first section (Table 1). In the second section, the respondents were asked about the highest-selling type of antimicrobials last month (Figure 2). The third, fourth, and fifth sections consisted of questions related to knowledge (nine questions), attitudes (six questions), and practices (eight questions) (Figure 3) related items, respectively. Both negative and positive items were included in each theme. The questionnaire was developed in English primarily and then translated into the local language, Bengali (Table S1). To check the accuracy of the translation, the Bengali version was translated back into English and compared with the preliminary version. Before administering the data collection, the questionnaire was pretested among a few numbers of drug and feed sellers to understand the suitability of the language. A slight modification was made based on the pretesting results to ensure the suitability of the language. However, the pretested interviews were excluded during the analysis. We collected data on two categories of closed-ended including ‘yes’ and ‘no’ questions on different knowledge, attitudes, and practices related to AMU and AMR. A two-point index (composite score range: 0–1) assigned values to responses for knowledge, attitude and practice items where correct response (“yes”) was assigned a value of 1, and incorrect response (“no”), 0.

### 2.4. Data Analyses

All the raw data were compiled, sorted, and imported to Microsoft Excel 2016 and analyzed using the statistical tool STATA/SE-16.1 (StataCorp, 4905, Lake Way Drive, College Station, TX 77845, USA). Cronbach’s alpha was used to measure the internal consistency of the questionnaire with an acceptable value of 0.76. To analyze the data, we used descriptive statistics, such as frequencies and percentages. Relationships between independent samples were explored using the chi-square test to determine if there were differences among respondents’ characteristics concerning the themes. Using the principal factor method described [34], we identified significant demographic characteristics and themes. The outcomes regarding knowledge, attitudes, and practices were categorized as “incorrect” versus “correct”; “negative” versus “positive,” and “bad” versus “good”, respectively. The distribution of individuals divided knowledge into two categories: less than 1 = incorrect knowledge and 1 = correct knowledge. Similar calculations were done for the items under attitudes and practices related to AMU and AMR.

Furthermore, this factor score analysis was also used as a part of the multivariate logistic regression analysis to determine the association with key themes regarding respondents’ demographics. The results are expressed as odds ratios (ORs) accompanied by 95% confidence intervals (95% CIs), and *p*-value < 0.05 was used as the threshold for statistical significance. Spearman’s rank-order correlation coefficient was used to describe the strength and direction of the relationship between responses to the knowledge, attitudes, and practices questions.

## 3. Results

We conducted 220 interviews for the current investigation (survey data are available in Table S1). The characteristics of the study interviewees are shown in Table 1. Out of the 220 interviews, 110 were carried out with drug and feed sellers equally. All of the respondents were male, and most of them belonged to the 31–35 years of age group (*n* = 63). In terms of years of experience, most of the respondents had 5–8 years of experience (*n* = 96), while most of the respondents had completed the 12th grade of education (*n* = 125). More than half of the respondents had not received any training on the drug (*n* = 113).

The most common class of antibiotics sold for use in poultry was Fluoroquinolone’s antibiotics, including ciprofloxacin (100% of drug sellers and 98.2% of feed sellers), enrofloxacin (80% of drug sellers and 49% of feed sellers), and oxytetracycline (92.7% of feed sellers and 74.6% of drug sellers), followed by penicillin (amoxicillin) (80% of feed sellers and 70% of drug sellers) (Figure 2).

### 3.1. Knowledge, Attitudes, and Practices of Poultry Drug and Feed Sellers on AMU and AMR

We asked nine questions to assess respondents’ knowledge of AMU and AMR. The results are shown in Figure 3. Most of the respondents (90% of drug sellers and 76% of feed sellers) said that antibiotics pass to humans from poultry; the drug sellers were more significantly (*p* < 0.05) reported this response. The feed sellers were more likely to say, “the whole flock requires treatment with antibiotics when one bird gets sick” (93% of all feed sellers) compared with drug sellers (70% of all drug sellers), which is statistically significant. All the drug sellers (100%) said antimicrobials have some side effects, while 89% of feed sellers reported a similar response. A larger proportion of the respondents (89% drug sellers and 82% feed sellers, respectively) did not know that antibiotics have similar curative effects on poultry, depicting correct knowledge. A sizeable proportion of respondents (53% of feed sellers and 30% of drug sellers) mentioned that antibiotics help in bacterial and viral infections, representing incorrect responses.

Interestingly, a quarter of the respondents (25% of drug and feed sellers, respectively) reported that antibiotics could be used for all diseases. The feed sellers were more significantly mentioned that they did not know about AMR compared with drug sellers (47% and 20%, respectively). Similarly, they were significantly mentioned that they did not aware of antimicrobial residue (47% and 30%, respectively). However, most of the respondents know about the withdrawal period of antibiotics (90% of drug sellers and 87% of feed sellers).

We asked six questions to the respondents to assess attitudes on AMR. The results are shown in Figure 3. Most of the respondents (85% of drug sellers and 73% of feed sellers) answered “negative” to the item ‘To prevent the wastage of antimicrobials, it is better to sell with less price when antibiotics are about to expire’. Although most of the respondents thought that the antibiotics should be placed in a restricted place, drug sellers were more significantly mentioned this response (80% and 60%, respectively). A sizeable proportion of respondents (45%) thought antimicrobials can be used with feed to prevent disease. However, the drug sellers significantly provided positive responses, when asking ‘missing a dose can lead to AMR’ compared with feed sellers (90% and 56%, respectively). Similarly, they were significantly more who thought that random use of antimicrobials can lead to AMR compared with feed sellers (80% and 56%, respectively).

We asked eight questions to assess respondents’ practices regarding AMU and AMR. The results are shown in Figure 3. A little less than 50% of all respondents (50% of drug sellers and 45% of feed sellers) mentioned the course of the antimicrobial drugs to the poultry farmers. The majority of the respondents (100% of drug sellers and 80% of feed sellers) reported that they encourage farmers not to eat poultry birds when they are at the final stage of antibiotics, depicting a good practice. More than half of the respondents (68% of drug sellers and 60% of feed sellers) reported, they do not randomly suggest antibiotics to the farmers. Feed sellers were more significantly mentioned that they do not stop using antibiotics when the poultry birds feel better than the drug sellers (71% and 58%, respectively).

Similarly, they significantly reported increases in the antimicrobials’ dose and frequency if the health conditions of the poultry birds do not improve (58% and 25%, respectively), representing a ‘bad’ practice. Nevertheless, the drug sellers were significantly encouraged to maintain withdrawal periods of antibiotics compared with feed sellers (79% and 48%, respectively). In terms of using antimicrobials as a growth promoter, most of the respondents (80% of drug sellers and 71% of feed sellers) did not perform this action, representing a ‘good’ practice. However, both drug and feed sellers sold antimicrobials without a prescription by the registered veterinarians (40% of drug sellers and 82% of feed sellers). The feed sellers were more significantly practiced this compared with the drug sellers.

### 3.2. Differences in Respondents’ Knowledge, Attitudes, and Practices

Principle factor analysis was performed to show the significant factors between the demographic variables and knowledge theme. The results are demonstrated in Table 2, showing that type of respondents (*p* = 0.000), respondents’ years of experience (*p* = 0.000), level of education (*p* = 0.000), and training on the drug (*p* = 0.000) were the significant factors influencing the knowledge theme. The analysis also revealed that the type of respondents (*p* = 0.000), respondents’ age (*p* = 0.047), and level of education (*p* = 0.003) were the significant factors affecting their attitudes.

The principal factor analysis of respondents’ practices demonstrated that respondents (*p* = 0.000) and years of experiences (*p* = 0.028) were the significant factors impacting their practices related to AMU and AMR.

The adjusted logistic regression analysis output on respondents’ demographic variables and their level of knowledge, attitudes, and practices is presented in Table 3. The results demonstrated that the drug sellers had 5.20 times the odds of having a proper knowledge of AMU and AMR (ORs = 5.20, CIs = 2.5–11.0, *p* = 0.00) compared with the feed sellers. The analysis also revealed that the respondents who had completed education up to 12th grade had 4.88 times the odds of having ‘correct’ knowledge than the respondents who completed graduation (ORs = 4.88, CIs = 2.42–9.85, 0.000, *p* = 0.000). Further, the respondents who received training on the drug had 5.22 times the odds of having ‘correct’ knowledge on AMU and AMR compared with their counterparts (ORs = 5.62, CIs = 2.52–10.81, *p* = 0.000). There was no significant variation found for the other variables.

In terms of attitudes, the results showed that the drug sellers had 26.71 times the odds of having a ‘better’ attitude towards AMU and AMR (ORs = 26.71, CIs = 10.5–68.0, *p* = 0.00) compared with the feed seller. They further revealed that the respondents who belong to the age group 31–35 and 36–40 years had a ‘better’ attitude (ORs = 3.72, CIs = 1.42–9.72, *p* = 0.007 and ORs = 4.59, CIs = 1.31–16.08, *p* = 0.017, respectively) compared with the respondents who were 41 years of age or more. Furthermore, the respondents who completed 12th grade had 7.80 times the odds of having ‘good’ attitudes than their counterparts (ORs = 7.80, CIs = 3.25–18.68, *p* = 0.000). There was no significant variation found for the other variables.

The analysis of AMU and AMR practices showed that the drug sellers had 14.16 times the odds of having a ‘better’ practice related to AMU and AMR (ORs = 14.16, CIs = 6.33–31.66, *p* = 0.00) compared with the feed sellers. It was observed that the respondents under the age categories of 18–25 and 26–30 years had ‘good’ practices related to AMU and AMR (ORs = 4.73, CIs = 1.33–16.85, *p* = 0.017 and OR = 5.85, CI = 2.03–16.87, *p* = 0.001) compared with the respondents who were 41 years of age or more. Interestingly, the respondents who completed 12th grade had 3.68 times the odds of performing ‘better’ practices (OR = 3.68, CI = 1.76–7.68, *p* = 0.001) compared with those who completed graduation. There was no significant variation observed for the other variables.

### 3.3. Relationship between Knowledge, Attitudes and Practices on AMU and AMR

Spearman’s rank-order correlation revealed a positive association between each pair of the knowledge, attitude, and practice scores for respondents (*p* ≤ 0.001). The correlation was fair between knowledge–attitudes, knowledge–practices, and attitudes–practices [35] (Table 4).

## 4. Discussion

Although it is less known that drug and feed sellers’ knowledge, attitude, and practices (KAP) influence the use of antimicrobials (AMU) and the level of antimicrobial resistance (AMR) in the poultry industry, to the best of our knowledge, this is the first known study conducted as such in Bangladesh. The study provided baseline evidence about KAPs regarding the suggestion of antimicrobials’ use in the poultry sector and offers insight into designing interventions to reduce antibiotic misuse. The current study further provides some significant insights for countries like Bangladesh with low-income settings. In Bangladesh, poultry farms are designed to receive feed and drugs directly from the feed and drug sellers [24]. Unfortunately, these are supplied through unprofessional sellers, in most instances without being prescribed by registered veterinarians [36]. They frequently suggest medicines without any foresight of the drugs owing to their inadequate knowledge and lack of expertise and skill. Therefore, this study was conducted to gain insight into the knowledge, attitude, and practices of the feed and drug sellers in the perspective of growing concerns about AMR. Both drug and feed sellers are front-liners and are responsible personnel for introducing irrational and indiscriminate antimicrobials practices into the poultry farm operation. Therefore, they should have sufficient and appropriate knowledge on AMU and AMR when suggesting antimicrobials [37].

The knowledge gap in understanding the emergence of bacterial resistance originating from poultry within resource-limited environments has been widely discussed [6,8,18,38,39,40]. A multi-country study in the African region showed that knowledge, attitudes, and particularly practices significantly varied by demographic factors such as age and years of experiences of the respondents [7]. Similarly, the current study has also observed a clear line of the knowledge gap among drug and feed sellers. The earlier study in the African region, although not among poultry drug and feed sellers, found that knowledge, attitudes, and particularly practices varied significantly by demographic factors such as age and years of experiences of the respondents [7]. The analysis of the current study showed consistency with earlier findings. In particular, the type of respondents (drug and feed sellers), years of experience, and training on the drug were the significant factors that dictate the application of antibiotics in the poultry industry.

In most cases, the feed sellers supplied antimicrobials to the farmers to obtain better revenue for the farm. Interestingly, the drug sellers had better knowledge of AMR than the feed sellers across all sets of parameters. The encouraging part of this study is that the drug sellers often receive suggestions about the completion of the dose of antimicrobials, and maintain the withdrawal periods before human consumption.

In contrast, the respondents with 12th grade education had far better knowledge of using drugs than their graduate counterparts. This is fascinating, but this could be the experience and eagerness about learning and implementing those in different situations. They usually receive information about drugs and their doses and schedules from registered veterinarians/medical representatives enrolled by different drug companies [41]. As we analyzed the training data among the respondents, we observed that those who had received training on drug use had higher knowledge of AMU and AMR compared with those who did not.

The study also showed that the respondents’ (drug and feed sellers), age, and education level were the significant factors in developing their attitude on the AMR issue. Similarly, in another study, it was found that respondents over 35 years of age were more likely to have a negative attitude towards AMU and AMR in the human and poultry interface [42]. Our study demonstrated that drug sellers showed positive attitudes regarding AMU and AMR as they have better knowledge. Considering the demographics variables, this scenario has been observed among those respondents who completed 12th grade of education compared with those who completed graduation. This could happen because of their expertise being developed over time and receiving different sorts of training and learning processes in different adverse conditions. The type of respondents and years of experience were significant factors in practices related to AMR. This study found that drug sellers had better practices of different antimicrobials and thus managed the AMU and AMR in better ways compared with the feed sellers, as they had better knowledge on these issues. The drug sellers might have different training on drug use and get more exposure through medical representatives as they are frequently selling their products in the same shops. This might enhance their knowledge and allow them to receive better suggestions from the representatives of different drug and feed companies [37].

However, both the drug and feed sellers usually discuss the profitability and better management practices with the farmers and suggest different types of non-antibiotics, including vitamins, and minerals [24]. The sad story of this unauthorized prescription that they might not suggest the withdrawal period of antimicrobials to all farmers, as this might maintain proper and precise tight schedules to reduce the AMR burden. The respondents who were 41 years or above had good practices of suggesting different antibiotics judiciously compared with the respondents who were 25 years or below. This is a usual phenomenon that plays a significant role in managing farmers through different ideas and suggestions on utilizing antibiotics. Those who are well experienced and have sufficient training and expertise may show good practices in delivering antibiotics to the farmers. They might have the ability to avoid unnecessary prescriptions on utilizing antibiotics.

Interestingly, the analysis has shown that the respondents who completed 12th grade were performing appropriate practices compared with the respondents who completed graduation. The drug and feed sellers mostly rely on their experiences and training other than institutional education in their work [43]. Those who have not completed graduation, but have had exposure in handling different cases, thus arguably have better experience from their workplace. As expected, the respondents who received training on the drug had better practices related to AMU and AMR compared with those who did not. The training on the drugs gives enough knowledge and instructions on AMU and the way of selling [43]. Those who have taken that kind of training can share their views with clients. This can be complemented by an interesting finding in a study in Vietnam which reported inappropriate practices of AMU among respondents, irrespective of educational attainment, though they had better knowledge and attitudes to AMR [44].

The study also identified a relationship between respondents having less knowledge, less favorable attitudes, and poor practices regarding the appropriate use of antimicrobials. These include groups such as those with lower formal education, who had less knowledge and less favorable attitudes and practices to AMU, and who could be targeted in different education campaigns. Aligned with other KAP studies on AMR [45,46,47], we found that the level of knowledge influenced the level of attitudes towards and practices of AMU and AMR. The correlation analysis of our study reported that respondents’ knowledge had a good relationship with their attitudes and practices. This finding indicated that increasing knowledge of AMR could shift the inappropriate practices related to AMU and AMR. However, owing to the nature of the study design, the current study cannot explain why these factors are significant predictors. Therefore, we strongly recommend further study that is preferably qualitatively exploratory to explain social and cultural perspectives.

The findings of this study offer several implications. Changing behavior is never easy, particularly where monetary profit is involved from the farmers’ and sellers’ perspective [48]. Since the National Action Plan on AMR (NAP AMR) of Bangladesh has acknowledged the importance of awareness raising, advocacy, communication, and developing continuous professional education curriculum from the ‘One Health’ approach [49], the poultry drug and feed sellers could be engaged with such initiatives. Our study provides an evidence base from which to develop educational programs for drug and feed sellers about antibiotic recommendations. For example, several respondents were performing inappropriate practices, which could potentially risk antibiotics being used in a similar way to other drugs, hence the need to increase knowledge on AMU and AMR and the ability to perform appropriate practices while making recommendations of antibiotics. The concept of AMR is known, but problems associated with antibiotic misuse were found to be imperfectly understood. The findings of the study also indicated that, when training on the drug is provided, the level of knowledge, attitudes, and appropriate practice increased, which also verifies the need for taking mass education campaigns. Therefore, targeting both of these groups is highly recommended.

## 5. Conclusions and Recommendations

There is a loop in antibiotic use as poultry drug and feed sellers provide credit and information for small-scale poultry farmers to initiate and operate their business in a profitable way. In return for credit, farmers are obliged to buy poultry feed and medicine from their sellers and sell their poultry products to the same seller. All farms applied multiple antimicrobials to poultry throughout the production cycle, including banned antimicrobials such as colistin sulfate. Therefore, educating farmers alone will not be enough to minimize any misuse of antimicrobials. A multi-faceted approach involving poultry drug and feed sellers using both educational and regulatory measures is needed. Such an approach should be embedded in a general policy to change the culture of antimicrobials’ use by improving awareness among frontline antibiotic sellers about the risks associated with AMU and reducing inappropriate recommendations of antibiotics for profit-making.

## Figures and Tables

**Table 1:** Demographic characteristics of the poultry drug and feed sellers.

Variables		*n* (%)
Type of respondents	Drug sellers	110 (50.0)
	Feed sellers	110 (50.0)
Age (Years)	Minimum–25	23 (10.5)
	26–30	42 (19.1)
	31–35	63 (28.6)
	36–40	35 (15.9)
	41 or more	57 (25.9)
Years of experience (Years)	0–4	25 (11.4)
	5–8	96 (43.6)
	9–12	79 (35.9)
	13 or more	20 (9.1)
Level of Education	Graduate	95 (43.2)
	Up to 12th grade	125 (56.8)
Training on drug	Non-trained	113 (51.4)
	Trained	107 (48.6)

**Table 2:** Test of statistical significance of variation in the respondents’ knowledge on AMU and AMR by their characteristics.

Variables		Knowledge			Attitudes			Practices		
		Incorrect: *n* (%)	Correct: *n* (%)	*p*	Negative: *n* (%)	Positive: *n* (%)	*p*	Bad: *n* (%)	Good: *n* (%)	*p*
Type of respondent	Drug sellers	45 (40.9)	65 (59.1)	0.000	27 (24.6)	83 (75.4)	0.000	33 (30)	77 (70)	0.000
	Feed sellers	72 (65.4)	38 (34.6)		84 (76.4)	26 (23.6)		84 (76.4)	26 (23.6)	
Age (years)	Minimum–25	12 (52.2)	11 (47.8)	0.074	14 (60.9)	9 (39.1)	0.047	11 (47.8)	12 (52.2)	0.077
	26–30	21 (50.0)	21 (50.0)		24 (57.1)	18 (42.9)		17 (40.5)	25 (59.5)	
	31–35	42 (66.7)	21 (33.3)		28 (44.4)	35 955.6)		37 (58.7)	26 (41.3)	
	36–40	13 (37.1)	22 (62.9)		11 (31.43)	24 (68.6)		15 (42.9)	20 (57.1)	
	41 or more	29 (50.9)	28 (49.1)		34 (59.6)	23 (40.4)		37 (64.9)	20 (35.1)	
Years of experience	0–4	20 (80.0)	5 (20.0)	0.000	16 (64.0)	9 (36.0)	0.078	18 (72.0)	7 (28.0)	0.028
	5–8	61 (63.5)	35 (36.5)		53 (55.2)	43 (44.8)		56 (58.3)	40 (41.7)	
	9–12	29 (36.7)	50 (63.3)		36 (45.6)	43 (54.4)		36 (45.6)	43 (54.4)	
	13 or more	7 (35.0)	13 (65.0)		6 (30.0)	14 (70.0)		7 (35.0)	13 (65.0)	
Level of Education	Graduate	65 (68.4)	30 (31.6)	0.000	59 (62.1)	36 (37.9)	0.003	60 (63.2)	35 (36.8)	0.673
	Up to 12th grade	52 (41.6)	73 (58.4)		52 (41.6)	73 (58.4)		57 (45.6)	68 (54.4)	
Training on drug	Non-trained	76 (67.3)	37 (32.7)	0.000	57 (50.4)	54 (50.5)	0.997	60 (53.1)	53 (46.9)	0.979
	Trained	41 (38.3)	66 (61.7)		56 (49.5)	53 (49.5)		57 (53.3)	50 (46.7)	

**Table 3:** Logistic regression analysis of the factors associated with respondents’ knowledge, attitudes, and practices on AMU and AMR.

Variables		Knowledge	Attitudes	Practices
		OR, 95%CI, *p*	OR, 95%CI, *p*	OR, 95%CI, *p*
Type of respondents	Feed sellers	Ref	Ref	Ref
	Drug sellers	5.20, 2.5–11.0, 0.000	26.71, 10.5–68.0, 0.000	14.16, 6.33–31.66, 0.000
Age (Years)	41 or more	Ref	Ref	Ref
	18–25	1.33, 0.42–4.22, 0.633	1.20, 0.30–4.71, 0.797	4.73, 1.33–16.85, 0.017
	26–30	1.24, 0.48–3.23, 0.658	1.52, 0.53–4.3, 0.435	5.85, 2.03–16.87, 0.001
	31–35	0.63, 0.25–1.56, 0.318	3.72, 1.42–9.72, 0.007	2.12, 0.84–5.36, 0.298
	36–40	1.26, 0.43–3.72, 0.672	4.59, 1.31–16.08, 0.017	2.58, 0.84–7.97, 0.098
Experience(Years)	0–4	Ref	Ref	Ref
	5–8	1.79, 0.54–5.93, 0.339	0.74, 0.24–2.31, 0.608	1.38, 0.45–4.21, 0.571
	9–12	3.78, 1.07–13.32, 0.039	0.64, 0.18–2.29, 0.493	1.80, 0.536.13, 0.348
	13 or more	3.23, 0.68–15.46, 0.142	1.60, 0.30–8.55, 0.578	2.89, 0.58–14.38, 0.196
Level of Education	Graduate	Ref	Ref	Ref
	Up to 12th grade	4.88, 2.42–9.85, 0.000	7.80, 3.25–18.68, 0.000	3.68, 1.76–7.68, 0.001
Training on drug	Not trained	Ref	Ref	Ref
	Trained	5.22, 2.52–10.81, 0.000	1.93, 0.90–4.15, 0.093	1.47, 0.72–2.98, 0.291

**Table 4:** Correlations between knowledge, attitudes, and practices on AMU and AMR.

Variables	Correlation Coefficient	*p*-Value
Knowledge–Attitudes	0.4731	0.000
Knowledge–Practice	0.3610	0.000
Attitudes–Practices	0.3456	0.000

## Data Availability

Survey data of knowledge, attitude, and practices on antimicrobial use and antimicrobial resistance among poultry drug and feed sellers in Bangladesh are given in Table S1.

## Supplementary Materials

The following are available online at https://www.mdpi.com/article/10.3390/vetsci8060111/s1, Table S1: Survey data of Knowledge, Attitude, and Practices on Antimicrobial Use and Antimicrobial Resistance among Poultry Drug and Feed Sellers in Bangladesh.
